# Diagnostic accuracy of staging laparoscopy for detecting metastasized or locally advanced perihilar cholangiocarcinoma: a systematic review and meta-analysis

**DOI:** 10.1007/s00464-016-4788-y

**Published:** 2016-02-19

**Authors:** Robert J. S. Coelen, Anthony T. Ruys, Marc G. H. Besselink, Olivier R. C. Busch, Thomas M. van Gulik

**Affiliations:** Department of Surgery, Academic Medical Center, Meibergdreef 9, 1105 AZ Amsterdam, The Netherlands

**Keywords:** Perihilar cholangiocarcinoma, Staging laparoscopy, Yield, Diagnostic accuracy, Resectability

## Abstract

**Background:**

Despite extensive preoperative staging, still almost half of patients with potentially resectable perihilar cholangiocarcinoma (PHC) have locally advanced or metastasized disease upon exploratory laparotomy. The value of routine staging laparoscopy (SL) in these patients remains unclear with varying results reported in the literature. The aim of the present systematic review was to provide an overview of studies on SL in PHC and to define its current role in preoperative staging.

**Methods:**

A systematic review and meta-analysis were performed in PubMed and EMBASE regarding studies providing data on the diagnostic accuracy of SL in PHC. Primary outcome measures were the overall yield and sensitivity to detect unresectable disease. Secondary outcomes were the yield and sensitivity for recent studies (after 2010) and large study cohorts (≥100 patients) and specific (metastatic) lesions. Methodological quality of studies was assessed with the Quality Assessment of Diagnostic Accuracy Studies tool.

**Results:**

From 173 records, 12 studies including 832 patients met the inclusion criteria. The yield of SL in PHC varied from 6.4 to 45.0 % with a pooled yield of 24.4 % [95 % confidence interval (CI) 16.4–33.4]. Sensitivity to detect unresectable disease ranged from 31.6 to 75 % with a pooled sensitivity of 52.2 % (95 % CI 47.1–57.2). Sensitivity was highest for peritoneal metastases (80.7 %, 95 % CI 70.9–88.3). Subgroup analysis revealed that the yield and sensitivity tended to be lower for studies after 2010. Considerable heterogeneity was detected among the studies.

**Conclusions:**

The results of the pooled analyses suggest that one in four patients with potentially resectable PHC benefits from SL. Given considerable heterogeneity, a trend to lower yield in more recent studies and further improvement of preoperative imaging over time, the routine use of SL seems discouraging. Studies that identify predictors of unresectability, that enable selection of patients who will benefit the most from this procedure, are needed.

**Electronic supplementary material:**

The online version of this article (doi:10.1007/s00464-016-4788-y) contains supplementary material, which is available to authorized users.

Perihilar cholangiocarcinoma (PHC) is a rare disease with a dismal prognosis [1, 2]. Radical surgery, consisting of a combined extrahepatic bile duct and partial liver resection, is the only curative treatment [3]. Despite various imaging techniques used for preoperative staging including state-of-the-art computed tomography (CT) or magnetic resonance imaging (MRI) scans, up to 47 % of patients have locally advanced or metastatic disease at surgical exploration [4, 5]. Staging laparoscopy (SL) prior to exploration may detect small liver metastases or peritoneal metastases that are frequently undetectable on routine CT or MRI scans. Additional SL may therefore prevent unnecessary laparotomy and associated postoperative morbidity or even mortality. However, the diagnostic yield of SL for PHC and its accuracy to detect unresectable disease remain unclear with varying results reported in the literature [6–8]. A recent study even found a decreasing diagnostic accuracy of SL over a period of 17 years in a single academic institution, possibly as a result of improved imaging techniques during the past decade [6]. As the place of routine SL in the preoperative staging of PHC is under debate, the aim of the present study was to define its current role by conducting a systematic review and meta-analysis of all available literature.

## Materials and methods

A systematic review was conducted by two independent authors (R.J.S.C. and A.T.R.) according to the Preferred Reporting Items for Systematic Reviews and Meta-Analyses (PRISMA) statement [9]. A study protocol was followed which defined the study objectives, eligibility criteria, outcome measures, search strategy and methodology of analysis (Appendix 1, ESM).

### Eligibility criteria

Both retrospective and prospective studies providing data on the diagnostic accuracy of SL (with or without additional laparoscopic ultrasound) in patients with PHC were considered for inclusion. Case reports, reviews, studies with less than 10 patients and patients with gallbladder carcinoma or intrahepatic cholangiocarcinoma were excluded. Findings at exploratory laparotomy and pathological examination were considered as reference standard for staging, except when laparoscopy detected biopsy-proven metastatic lesions, locally advanced tumors or benign disease.

### Outcome measures

Primary outcome measures were the overall yield of SL and diagnostic accuracy in terms of sensitivity to detect unresectable disease. The yield represents the number of patients (expressed as a percentage of all patients that undergo SL) that are withheld from an unnecessary laparotomy. The yield thereby reflects the proportion of patients that benefit from the SL procedure. Specificity was not considered as an endpoint as the specificity of SL is always 100 % since there are no false positives; laparoscopy and the reference standard are the same if histological examination during SL is positive.

As secondary analysis, the yield and sensitivity were calculated for more recent studies (after 2010) and studies with at least 100 patients. Also, sensitivity was separately investigated for combined liver and peritoneal metastases, liver metastases only, peritoneal metastases only, lymph node metastases and locally advanced disease (tumor invading vascular structures or surrounding organs). The additional diagnostic value of intraoperative ultrasound (IOUS) during SL was also investigated.

### Search strategy

A literature search was conducted in PubMed and EMBASE using MeSH and free text words with the aid of a clinical librarian. No language or time period restrictions were applied. Two reviewers (R.J.S.C. and A.T.R.) independently screened for relevance in titles and abstracts retrieved from the search. Selected articles were then assessed in full length by both reviewers to check the eligibility criteria. Disagreements during the search and selection process were resolved by discussion, and when needed, a third author (T.M.v.G.) was asked. The reference lists of eligible articles were checked for additional fitting papers. The search was updated until August 1, 2015.

#### Search in PubMed and EMBASE:

PubMed: (“Cholangiocarcinoma”[MeSH] OR “Bile Duct Neoplasms”[Mesh] OR “Klatskin’s Tumor”[Mesh] OR cholangiocarcinoma*[tiab] OR klatskin tumor*[tiab] OR klatskin tumour*[tiab] OR HCCA[tiab] OR proximal bile duct tumor* OR extrahepatic bile duct tumor* OR proximal bile duct cancer* OR extrahepatic bile duct cancer* OR proximal bile duct tumour* OR extrahepatic bile duct tumour* OR proximal biliary cancer*) AND (“Laparoscopy”[MeSH Terms] OR laparoscop*[tiab]) AND (“Neoplasm Staging”[Mesh] OR staging[tiab]).

#### EMBASE:

(exp bile duct carcinoma/or exp bile duct tumor/or Klatskin tumor/or (cholangiocarcinoma* or klatskin tumor* or klatskin tumour* or HCCA or proximal bile duct tumor* or extrahepatic bile duct tumor* or proximal bile duct cancer* or extrahepatic bile duct cancer* or proximal bile duct tumour* or extrahepatic bile duct tumour* or proximal biliary cancer* OR bile tract carcinoma* OR biliary carcinoma* OR biliary duct carcinoma* OR biliary tract carcinoma*).ti,ab,kw.) AND (exp laparoscopy/or laparoscop*.ti,ab,kw.) AND (cancer staging/or staging/or staging.ti,ab,kw.).

### Data collection

Data were independently extracted by the two reviewers using a spreadsheet. Data were collected taking into account author and institution, publication date, study period, study design, number of patients undergoing laparoscopy, number of completed procedures, number of patients undergoing laparoscopic intraoperative ultrasound (IOUS), number of avoided laparotomies, total number of patients with unresectable disease, number of liver, peritoneal and lymph node metastases, locally advanced disease, other reasons for unresectability, true positives, true negatives, false positives, false negatives, complications following SL and time interval between SL and laparotomy. Previously reported smaller series by the same institution in the same study period were considered as duplicates and excluded.

The yield was calculated by dividing the total number of avoided laparotomies by the total number of laparoscopies. Sensitivity was calculated by dividing the total number of avoided laparotomies by all patients with unresectable disease. Likewise, for calculating the sensitivity of SL for detecting specific lesions (e.g., peritoneal metastases) the total number of those specific lesions detected by SL was divided by all of those specific lesions at both SL and laparotomy.

### Quality assessment

Methodological quality of included studies was independently assessed by the two reviewers using the Quality Assessment of Diagnostic Accuracy Studies (QUADAS-2) tool, which is specifically developed for systematic reviews of diagnostic accuracy studies [10]. Risk of bias and applicability concerns were assessed for each included study using this tool, which is integrated in the RevMan software (Review Manager version 5.3. Copenhagen: The Nordic Cochrane Centre, The Cochrane Collaboration, 2014).

### Statistical analysis

Yield and diagnostic accuracy results from individual studies were graphically presented by plotting the sensitivity estimates [with 95 % confidence intervals (CIs)] using StatsDirect version 2.8.0 (StatsDirect statistical software. http://www.statsdirect.com. England: StatsDirect Ltd. 2013) and Meta-DiSc version 1.4 (XI Cochrane Colloquium, Barcelona, 2006), respectively. Heterogeneity among studies was tested using Cochran’s Q-test, and the amount of variation by heterogeneity was reflected by the inconsistency index value (*I*^2^). A *I*^2^ value above 75 % was considered as substantial heterogeneity. Results of individual studies were pooled, and summary estimates were calculated using a random-effect model (DerSimonian–Laird) in all cases. Individual studies lacking data to obtain sensitivity rates were excluded from meta-analysis.

## Results

### Search results

**Fig. 1:**
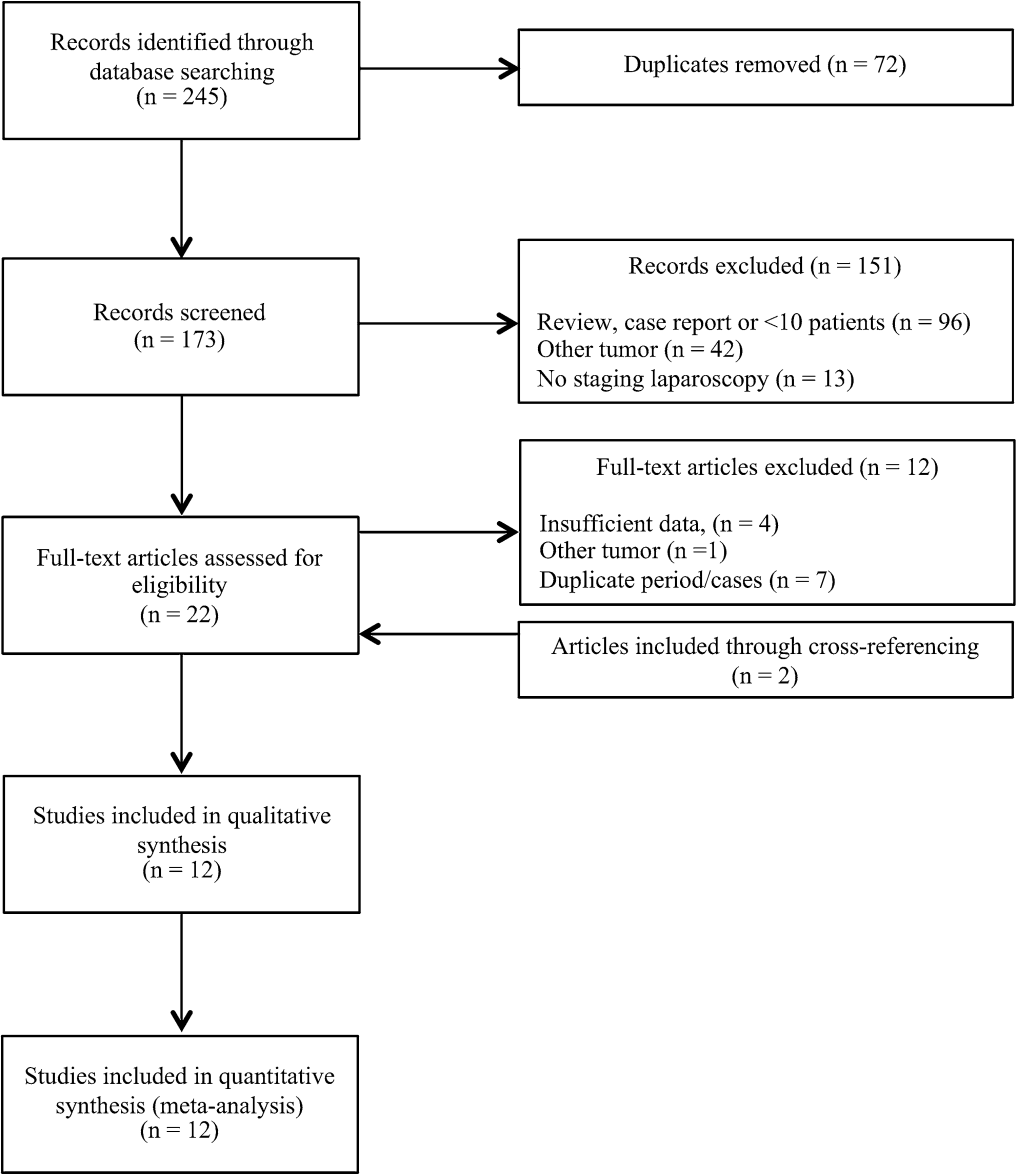
Flowchart of study selection process

One hundred and seventy-three records, excluding duplicates, were retrieved from the electronic database search. A total of 12 studies were included in the analysis. Figure 1 shows a flowchart of the search and selection process.

### Study characteristics

Eleven retrospective studies and one prospective study were identified providing an all-Western population of 832 PHC patients who had undergone SL between 1992 and 2014 (Table 1). The individual study population ranged from 10 patients in the smallest series to 175 in the largest and one of the more recent series. Except for one study in which 6 patients were included that appeared to have unresectable gallbladder carcinoma (3 at SL and 3 at laparotomy) [11], all studies provided specific data from a PHC-only study cohort. Almost all SL procedures (98 %) were completed (Table 1). Four studies reported morbidity rates (0–3 %) following SL, and the exact time interval between SL and laparotomy was reported in only three studies (range 6.5–37 days). One study described that almost all patients had undergone laparotomy immediately following SL [7]. Laparoscopic IOUS, whether performed in all patients or not, was reported in 9 studies (Table 2). Table 3 provides an overview of preoperative imaging performed in each study.

**Table 1:** Characteristics of included studies, listed according to time period

Study	Country	Time period	Study type	Patients undergoing SL, *n*		Morbidity, *n* (%)	Median interval SL laparotomy (days)
				Total	Completed		
Vollmer et al. [15]	USA	1996–1999	Retrospective	23	23	0	–
Tilleman et al. [11]^a^	Netherlands	1993–2000	Retrospective	110	107	3 (3 %)	36
Weber et al. [7]	USA	1997–2001	Retrospective	56	56	–	–
Rodgers et al. [26]	New Zealand	1999–2001	Prospective	10	–	–	–
Silva et al. [27]	UK	1992–2003	Retrospective	25	–	–	–
Connor et al. [13]	UK	1992–2003	Retrospective	83	79	–	–
Goere et al. [28]	France	2002–2004	Retrospective	20	19	–	–
Ruys et al. [6]	Netherlands	2000–2010	Retrospective	175	175	5 (3 %)	37
Barlow et al. [12]	UK	1998–2011	Retrospective	100	99	2 (2 %)	6.5
Gomez et al. [29]	UK	2001–2012	Retrospective	101	101	–	–
Ratti et al. [16]	Italy	2004–2012	Retrospective	94	89	–	–
Russolillo et al. [14]	Italy	2006–2014	Retrospective	35	35	–	–
Total				832	783/797 (98 %)		

*SL* staging laparoscopy—data not available

^a^Six patients were identified with unresectable gallbladder carcinoma (3 during SL and 3 at laparotomy)

**Table 2:** Overview of yield and sensitivity of SL among included studies

Study	Time period	Patients undergoing SL, *n*			Unresectable at SL	Yield (%)	Patients planned for laparotomy, *n*		
		Total	Completed	IOUS, *n*			Cancelled	Unresectable	Sensitivity (%)
Vollmer et al. [15]	1996–1999	23	23	Yes, 17	4	17.4	0	3	57.1
Tilleman et al. [11]	1993–2000	110	107	Yes, 74	44	40.0	1	30	59.5
Weber et al. [7]	1997–2001	56	56	Yes, –	14	25.0	0	19	42.4
Rodgers et al. [26]	1999–2001	10	–	Yes, –	3	30.0	–	1	75.0
Silva et al. [27]	1992–2003	25	–	–	5	20.0	0	–	–
Connor et al. [13]	1992–2003	83	79	Yes, all	35	42.2	9	19	64.8
Goere et al. [28]	2002–2004	20	19	No	5	25.0	–	6	45.5
Ruys et al. [6]	2000–2010	175	175	Yes, 4	24	13.7	10	52	31.6
Barlow et al. [12]	1998–2011	100	99	Yes, –	45	45.0	5	18	71.4
Gomez et al. [29]	2001–2012	101	101	No	18	17.8	0	26	40.9
Ratti et al. [16]	2004–2012	94	89	Yes, –	6	6.4	0	8	42.9
Russolillo et al. [14]	2006–2014	35	35	Yes, all	5	14.3	3	4	55.6

*SL* staging laparoscopy—data not available, *IOUS* intraoperative ultrasound

Yield calculated by dividing number of unresectable cases at SL by total number of SL procedures

Sensitivity calculated by dividing number of unresectable cases at SL by total number of unresectable cases

**Table 3:** Overview of preoperative imaging among included studies

Study	Yield (%)	Sensitivity (%)	Preoperative imaging						
			US	US duplex	EUS	CT	MRI	PET	CT/MRI technique
Vollmer et al. [15]	17.4	57.1	−	+	−	+	−	−	Triple-phase helical CT, 3-mm slice thickness
Tilleman et al. [11]	40.0	59.5	−	+	Selectively	+	Selectively	−	Before 1995 conventional CT, after 1995 spiral CT
Weber et al. [7]	25.0	42.4	NR	NR	NR	NR	NR	NR	NR
Rodgers et al. [26]	30.0	75.0	−	−	−	−	+	−	MR arteriography and cholangiography
Silva et al. [27]	20.0	−	−	+	−	+	+	−	High-resolution spiral CT, MRCP
Connor et al. [13]	42.2	64.8	+	−	−	+	Selectively	−	Selective additional arteriography
Goere et al. [28]	25.0	45.5	−	−	−	+	+	−	Triple-phase CT, selective additional arteriography
Ruys et al. [6]	13.7	31.6	+	+	−	+	Selectively	Selectively	NR
Barlow et al. [12]	45.0	71.4	+	−	−	+	Selectively	−	NR
Gomez et al. [29]	17.8	40.9	−	−	−	+	+	−	Triple-phase CT, MRI from 2008
Ratti et al. [16]	6.4	42.9	−	−	−	+	+	Selectively	NR
Russolillo et al. [14]	14.3	55.6	+	−	Selectively	+	+	Selectively	NR

*US* ultrasound, *US duplex* ultrasound with color flow Doppler, *EUS* endoscopic ultrasound, *CT* computed tomography, *MRI* magnetic resonance imaging, *PET* positron emission tomography, *NR* not reported

### Methodological quality of included studies

**Fig. 2:**
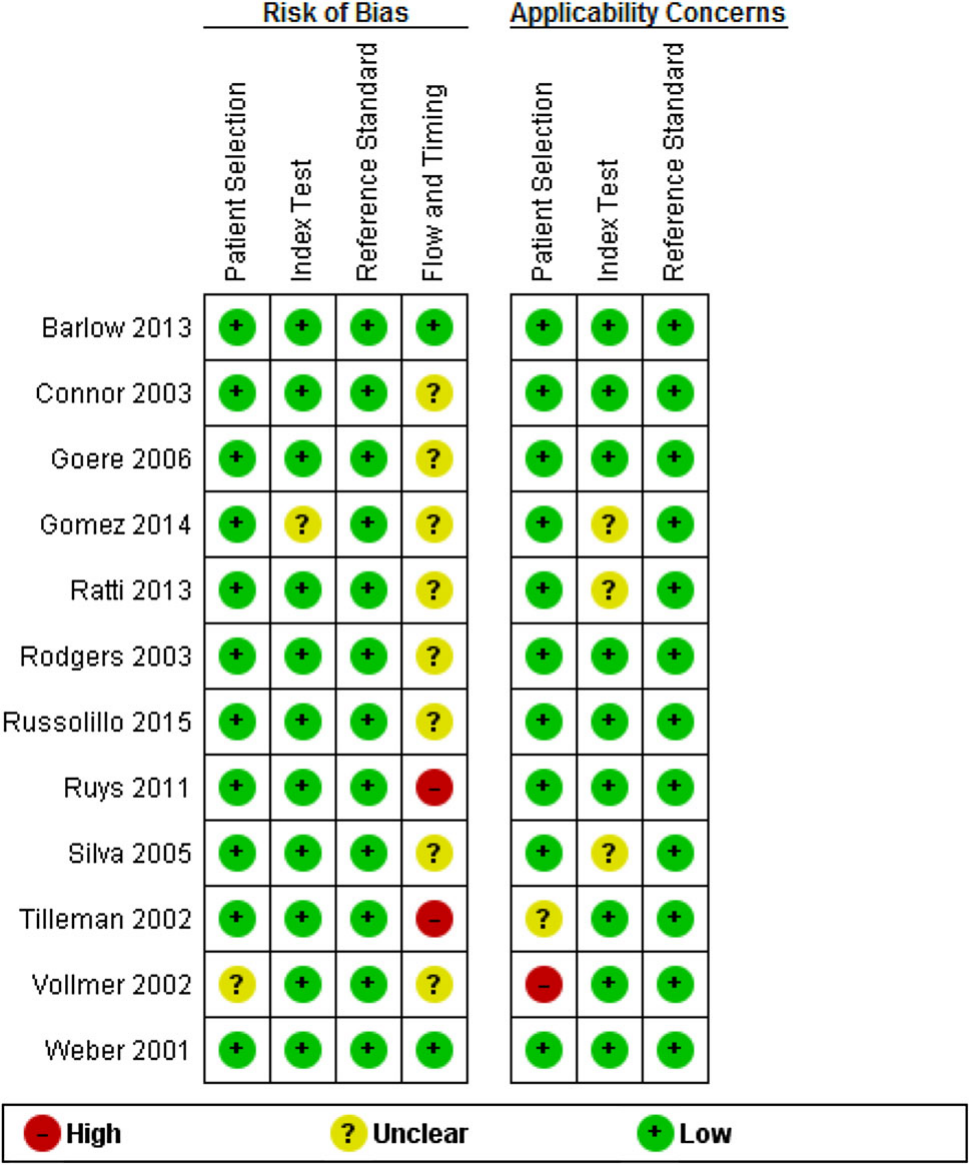
Risk of bias and applicability concerns for each included study

**Fig. 3:**
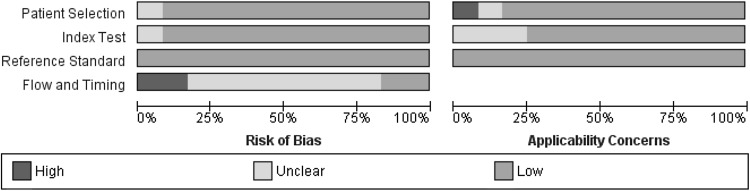
Risk of bias and applicability concerns presented as percentages across the included studies

Results of the quality assessment of included studies using the QUADAS-2 tool are presented in Figs. 2 and 3. Two studies were judged as low risk of bias and low applicability concern in all domains [7, 12]. The SL procedure was well described in most studies. Two studies were judged as high risk of bias regarding flow and timing as the interval between index test (SL) and reference test (explorative laparotomy) was relatively long compared to other studies, although most studies did not report this time interval [6, 11].

### Synthesis of outcome

**Fig. 4:**
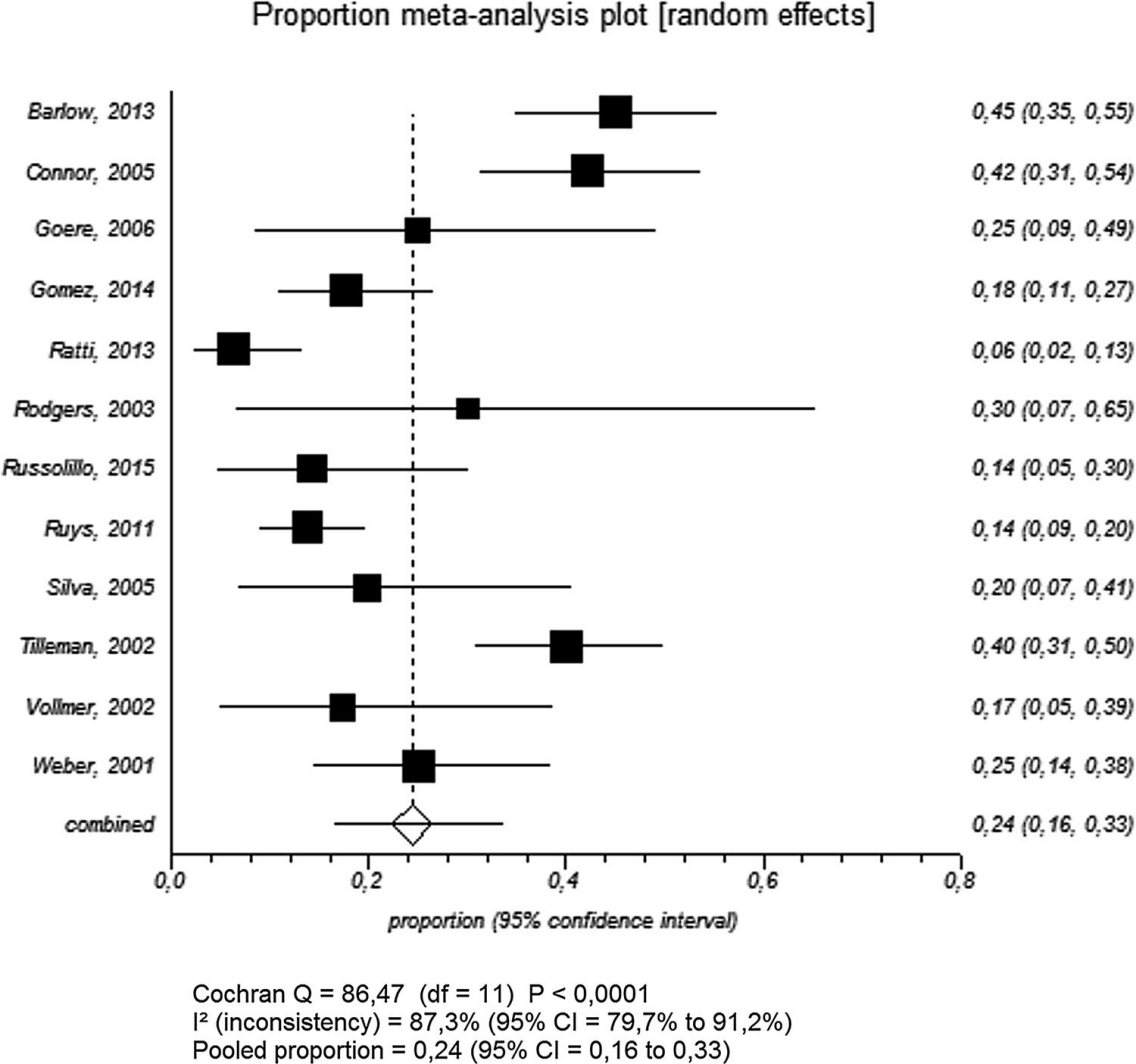
Meta-analysis of yield of SL in PHC among included studies

The yield of SL in patients with potentially resectable PHC varied from 6.4 to 45.0 % among the 11 studies (Table 2). Pooled yield was 24.4 % (95 % CI 16.4–33.4) with a high level of heterogeneity (*I*^2^ = 87.3 %; Fig. 4). Subgroup analysis revealed that the yield was lower for studies published beyond 2010 (18.6, 95 % CI 8.1–32.2, *I*^2^ = 91.9 %) and slightly higher for studies with more than 100 patients (28.2, 95 % CI 14.0–45.1, *I*^2^ = 93.5; Table 4).

**Table 4:** Subgroup analysis

	Sensitivity, % (95 % CI)	*I* ^2^ (%)	Yield, % (95 % CI)	*I* ^2^ (%)
*Overall results*	52.2 (47.1–57.2)	69.8	24.4 (16.4–33.4)	87.3
Studies after 2010	47.6 (40.6–54.6)	83.3	18.6 (8.1–32.2)	91.9
Studies ≥ 100 patients	51.0 (44.7–57.2)	88.7	28.2 (14.0–45.1)	93.5
All liver and peritoneal metastases	76.7 (69.4–82.9)	49.4	NA	
Liver metastases	59.0 (42.1–74.4)	40.3	NA	
Peritoneal metastases	80.7 (70.9–88.3)	59.0	NA	
Nodal metastases	58.8 (32.9–81.6)	74.8	NA	
Locally advanced	32.6 (23.2–43.2)	84.6	NA	

*NA* not applicable, *CI* confidence interval

All tests showed significant heterogeneity, except the pooled sensitivity of liver metastases (*P* = 0.14) and combined liver and peritoneal metastases (*P* = 0.054)

**Fig. 5:**
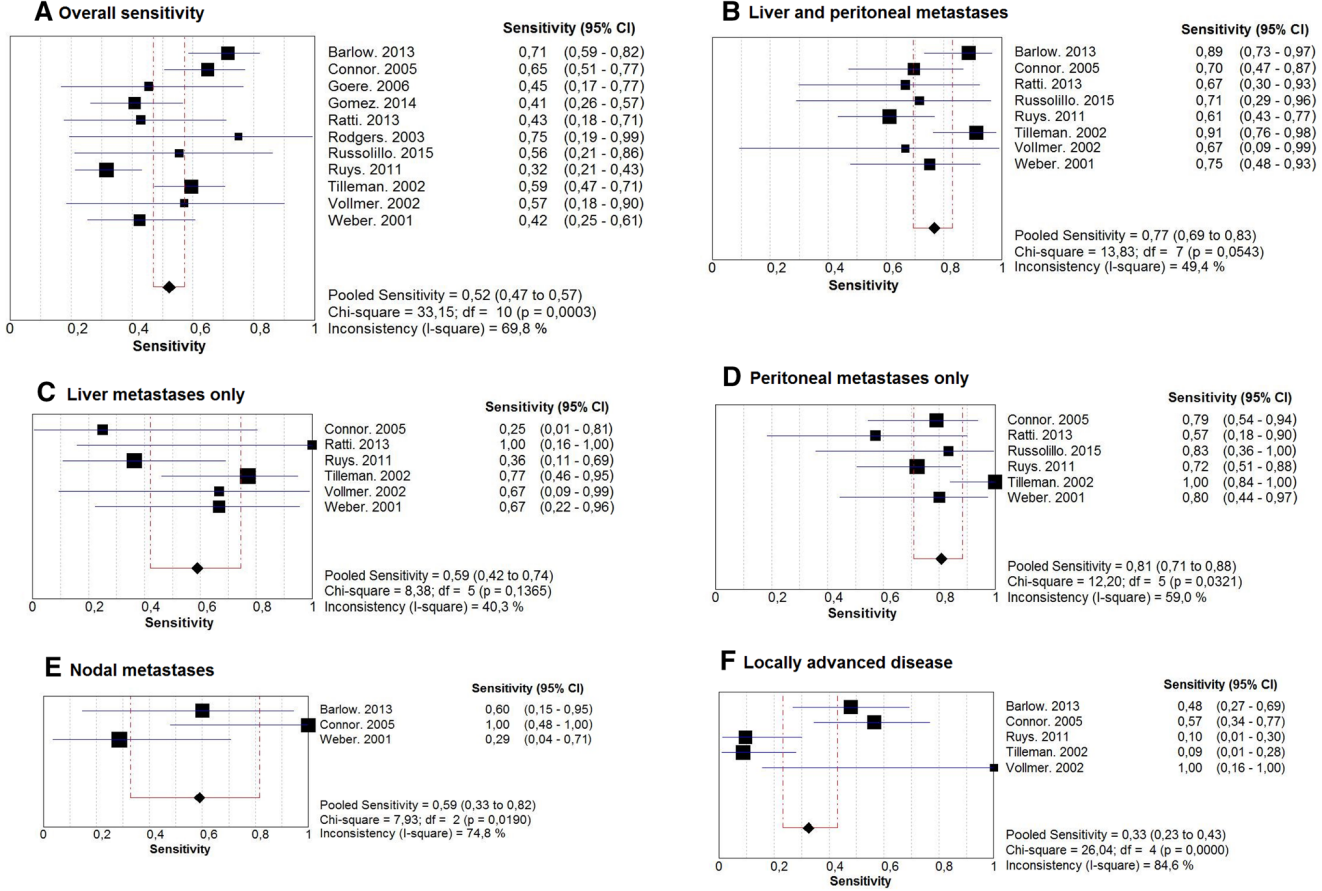
Meta-analysis of sensitivity of SL to detect unresectable disease and specific lesions in PHC among included studies

The diagnostic accuracy of SL in terms of overall sensitivity to detect unresectable disease could be retrieved from 11 of 12 studies and varied from 31.6 to 75.0 % (Table 2). The pooled sensitivity was 52.2 % (95 % CI 47.1–57.2) with a moderate level of heterogeneity (*I*^2^ = 69.8 %; Fig. 5A). Data on liver and peritoneal metastases were provided by 7 studies, whereas 8 studies specified the number of nodal metastases and locally advanced disease (Table 5). Staging laparoscopy had the highest sensitivity to detect peritoneal metastases (80.7 %, 95 % CI 70.9–88.3, *I*^2^ = 59.0 %), and the sensitivity for liver metastases was 59.0 % (95 % CI 42.1–74.4, *I*^2^ = 40.3 %; Fig. 5C, D). The pooled sensitivity for both liver and peritoneal metastases among 8 of 12 studies was 76.7 % (95 % CI 69.4–82.9, *I*^2^ = 49.4 %; Fig. 5B). The overall sensitivity to detect unresectable disease in studies beyond 2010 tended to be lower, whereas a less clear difference was seen for studies with more than 100 patients. Results of all subgroup analysis are shown in Table 4.

**Table 5:** Reasons for unresectability at SL and laparotomy among included studies

Study	Patients	Reason unresectable at SL					Reason unresectable at laparotomy (% accuracy)				
		Liver metastases	Peritoneal metastases	Nodal metastases	Locally advanced	Other^a^	Liver metastases	Peritoneal metastases	Nodal metastases	Locally advanced	Other^a^
Vollmer et al. [15]	23	2	0	0	2	0	1 (66.7)	0 (–)	2 (0)	0 (100)	0
Tilleman et al. [11]	110	10	21	0	2	0	3 (76.9)	0 (100)	2 (0)	21 (8.7)	4
Weber et al. [7]	56	4	8	2	0	0	2 (66.7)	2 (80.0)	5 (28.6)	10 (0)	0
Rodgers et al. [26]	10	–	–	–	–	–	–	–	–	–	–
Silva et al. [27]	25	–	–	–	–	–	–	–	–	–	–
Connor et al. [13]	83	1	15	5	13	1	3 (25.0)	4 (78.9)	0 (100)	10 (56.5)	2
Goere et al. [28]	20	–	–	–	–	–	–	–	–	–	–
Ruys et al. [6]	175	4	18	0	2	0	7 (36.4)	7 (72.0)	19 (0)	19 (9.5)	0
Barlow et al. [12]	100	31^b^	–	3	11	0	2 (–)	2 (–)	2 (60.0)	12 (47.8)	0
Gomez et al. [29]^c^	101	–	–	–	–	–	0 (100.0)	0 (100.0)	0 (–)	26 (–)	0
Ratti et al. [16]	94	2	4	0	0	0	0 (100.0)	3 (57.1)	0 (–)	5 (0)	0
Russolillo et al. [14]	35	0	5	0	0	0	1 (0)	1 (83.3)	0 (–)	2 (0)	0

*SL* staging laparoscopy—data not available

Accuracy calculated by dividing number of unresectable cases at SL by total number of unresectable cases

^a^Other reasons such as severe cirrhosis, benign disease or unspecified

^b^Number of liver and peritoneal metastases specified was not available, and overall accuracy to detect liver/peritoneal metastases was 31/35 (88.6 %)

^c^Eighteen patients in this study were unresectable at SL (either because of metastases or locally advanced disease)

The additional value of IOUS during SL could be evaluated from 6 studies. In the remaining 3 studies, insufficient data were available to investigate the additional value. In only two of the 6 studies, all patients had undergone laparoscopic IOUS [13, 14]. Two studies showed beneficial value of IOUS. In one of the studies where all patients were subjected to IOUS during SL, the yield increased with 17 % with the use of IOUS, which was mainly due to identification of locally advanced disease [13]. Only one patient in this study had deep liver metastases detected by IOUS. In another study, 50 % (2/4) of unresectable lesions were identified by IOUS and these were locally advanced tumors [15]. In the remaining studies that provided data on IOUS, no additional value was found as only 1 patient from these studies was detected with unresectable disease who did not also have unresectable disease on laparoscopic inspection alone [7, 11, 14, 16]. Furthermore, in two of these studies, all locally advanced tumors were missed at laparoscopic ultrasonography assessment [14, 16]. Due to lack of most data, pooling of IOUS results could not be performed.

## Discussion

From 12 studies reporting on the additional value of SL in 832 patients with PHC, we demonstrated that in 1 out of 4 patients undergoing SL, an unnecessary laparotomy was avoided. The overall sensitivity of SL to detect unresectability was relatively low (52 %), but subgroup analysis revealed that sensitivity for liver and peritoneal metastases was reasonable (77 %). The latter could be explained by a good sensitivity of SL for detecting peritoneal metastases only (81 %).

Despite extensive preoperative staging, still almost half of patients appear to have locally advanced tumors or metastases (liver, peritoneal or nodal) upon exploratory laparotomy. Liver and peritoneal metastases are readily detectable at SL, as was shown in our meta-analysis, but they are the reason for unresectability in only up to a third of all PHC cases [4]. Most studies in this systematic review showed poor accuracy to detect locally advanced disease or nodal metastases. These numbers explain why only a subset of patients actually benefits from this additional procedure. Furthermore, preoperative imaging with CT and MRI has drastically improved in the last decade making it easier to detect (extra)hepatic metastases, lymph node metastases and locally advanced disease [17]. Only smaller lesions may remain that are also easily missed at SL. Accordingly, our analysis showed that the yield and sensitivity in studies published after 2010 tended to be lower. Preoperative imaging was more extensive in these studies. So far, only two studies have shown any factors (i.e., Blumgart T-stage) that increase the diagnostic yield in these patients [7, 13], whereas several other predictors (e.g., CA19-9, tumor size) of unresectable disease at SL have been identified and validated for pancreatic cancer patients [18–23].

The value of IOUS during SL seems questionable as only the minority of studies reported any additional value in detecting local unresectability and metastatic disease. Laparoscopic ultrasound may identify less superficial liver metastases or tumor involvement of branches of the portal vein or hepatic artery, which would be impossible without exploration of the hilum, but the studies in this review provided insufficient data supporting its added value.

Ideally, SL and laparotomy are performed in a single session, to avoid two hospital admissions and two surgical procedures. In many centers, however, SL is performed separately from subsequent laparotomy due to logistical reasons related to anesthetic and operating room time planning. Furthermore, it is convenient to perform SL in an early phase as rapid detection of unresectability allows timely start of palliative care (metal biliary stents and chemotherapy). Detection of unresectability at laparotomy would otherwise require time for adequate biliary drainage and/or time for hypertrophy to occur after portal vein embolization. Two studies in our systematic review, however, reported a median interval time between SL and laparotomy of more than 5 weeks in which time small undetected lesions potentially have become large enough to be detected at laparotomy. This may partly explain a low sensitivity in one of these studies [6]. Another reason to perform SL prior to laparotomy in separate sessions is that assessment of biopsies may not be conclusive on frozen-section examination and require time for definitive histopathological diagnosis.

A previous systematic review on staging laparoscopy in proximal bile duct tumors also included a substantial number of gallbladder cancer patients from these series and even studies with gallbladder carcinomas only [8]. As gallbladder carcinomas are considered as a distinct entity that more frequently metastasizes to the liver or peritoneum, adding these patients to the current meta-analysis would lead to overestimating of the diagnostic value of SL in PHC [24, 25]. Moreover, our updated analysis on the topic includes five large recent studies comprising more than half of the total review cohort. We did include one large study in a PHC cohort in which three patients appeared to have an unresectable gallbladder carcinoma at SL and three patients at laparotomy [11]. As it was not possible to exclude only these six patients from analysis, we chose not to exclude the whole study as these patients had been diagnosed on preoperative imaging as having a PHC, which reflects clinical practice. In another study included in our analysis, the performance of a pancreaticoduodenectomy in some cases may suggest distal cholangiocarcinomas that were preoperatively diagnosed as Bismuth type 1 or 2 perihilar tumors [15].

Several limitations may apply to our meta-analysis. Firstly, the number of studies that could be included in the review was low. Relatively few centers worldwide have extensive experience with the surgical management of PHC and not every center routinely performs SL for these tumors. Remarkably, we analyzed an all-Western study population and no studies were retrieved from Asia where the highest incidence of PHC is found. Secondly and most importantly, our meta-analysis showed a significant amount of heterogeneity. The variation in outcomes between the studies was probably caused by differences in cohort size, time interval between SL and laparotomy and time period (only 5 study cohorts included patients after 2010). Nonsignificant, moderate heterogeneity was observed for pooled estimates of sensitivity to detect combined liver and peritoneal metastases and liver metastases only. Significant heterogeneity for most other outcomes persisted even after performing subgroup analyses in studies with more than 100 patients or studies published after 2010. Unfortunately, some studies could not be included in the sensitivity subgroup analysis as some data were unavailable in order to allow for profound analysis. This may have biased the sensitivity rates to detect liver and peritoneal metastases. Apart from possible flaws in flow and timing of the index test (SL) among studies, as mentioned previously, the overall methodological quality of studies assessed with the QUADAS-2 tool was reasonable.

In conclusion, results from this systematic review suggest that 1 in 4 patients with PHC benefits from SL with the highest sensitivity particularly for detecting peritoneal metastases. However, due to considerable heterogeneity among available studies, pooled estimates should be carefully interpreted. As the yield and sensitivity of SL may decrease over years with further improvement of preoperative imaging techniques, the utility of this additional staging modality may further diminish, thereby discouraging its routine use. Large studies that identify predictors of unresectable disease at SL, that can be used to select PHC patients who may benefit most from this procedure, are warranted.

## Electronic supplementary material

Below is the link to the electronic supplementary material.

Supplementary material 1 (DOCX 30 kb)
