# Supplementary material for: Diagnostic accuracy of staging laparoscopy for detecting metastasized or locally advanced perihilar cholangiocarcinoma: a systematic review and meta-analysis
Source: Surg Endosc. 2016 Feb 19;30(10):4163–73. doi: 10.1007/s00464-016-4788-y (PMC5009158; doi:10.1007/s00464-016-4788-y)
Supplement: Supplementary file 1 — Supplementary material 1 (DOCX 30 kb) [file 464_2016_4788_MOESM1_ESM.docx]

**Appendix 1. Systematic review study protocol (July 2015)**

**Authors**

R.J.S. Coelen, A.T. Ruys, M.G. Besselink, O.R.C. Busch, T.M. van Gulik

Department of Surgery, Academic Medical Center

**Title**

Staging laparoscopy for detecting unresectable disease in patients with potentially resectable perihilar cholangiocarcinoma: a systematic review and meta-analysis.

**Background**

Perihilar cholangiocarcinoma (PHC) is a rare disease with a dismal prognosis. Radical surgery, consisting of a combined extrahepatic bile duct and partial liver resection, is the only curative treatment^[1]^. Despite various imaging techniques used for preoperative staging including state-of-the-art computed tomography (CT) or magnetic resonance imaging (MRI) scans, up to 47% of patients have locally advanced or metastatic disease at surgical exploration^[2, 3]^. Staging laparoscopy (SL) may prevent unnecessary laparotomy and associated postoperative morbidity and even mortality. However, the diagnostic yield of routine SL for PHC remains unclear with varying results reported in literature.

The aim of the present study is to conduct a systematic review providing an overview of reports on SL in PHC and to define its current role in preoperative staging.

**Objectives**

To perform a systematic review and meta-analysis concerning the diagnostic yield and sensitivity of staging laparoscopy for potentially resectable perihilar cholangiocarcinoma.

**Outcomes**

*Primary outcome*

- Overall yield: the number of patients (expressed as a percentage of all patients that undergo SL) that are withheld from an unnecessary laparotomy
- Diagnostic accuracy (sensitivity) to detect unresectable disease

*Secondary outcome*

- Yield and sensitivity for studies after 2010 and ≥ 100 patients
- Sensitivity for specific lesions:
  - Combined liver and peritoneal metastases
  - Liver metastases only
  - Peritoneal metastases only
  - Lymph node metastases
  - Locally advanced disease
- Additional diagnostic value of laparoscopic intraoperative ultrasound (IOUS)

**Methods**

For this review, the Preferred Reporting Items for Systematic Reviews and Meta-Analyses (PRISMA) guidelines will be applied^[4]^. The objectives, search methodology and eligibility criteria are pre-specified in the current protocol.

*Eligibility criteria*

This systematic review will include all retrospective and prospective studies that have examined the effect of SL (with or without additional laparoscopic IOUS) on the surgical management of patients with potentially resectable PHC based on preoperative imaging. Findings at exploratory laparotomy and pathological examination are considered reference standard for staging, except when laparoscopy detected biopsy-proven metastatic lesions, locally advanced tumors or benign disease.

Case reports, reviews, studies with less than 10 patients, duplicates and patients with gallbladder carcinoma or intrahepatic cholangiocarcinoma will be excluded from analysis.

*Search strategy*

A clinical librarian will help with the search strategy. MeSH and free text words concerning staging laparoscopy and perihilar cholangiocarcinoma will be used. No language or time period restrictions will be applied. The literature search is conducted in PubMed and EMBASE (provider Ovid). Two authors (R.J.S.C. and A.T.R.) will independently screen for relevance in titles and abstracts retrieved from the search. The reference lists of eligible articles will be checked for additional fitting papers. Selected articles will be assessed in full length by both reviewers to check the eligibility criteria. Disagreements during the search and selection process will be resolved by discussion and, if needed, a third author (T.M.v.G.) will decide. The complete search is shown below.

PubMed: ("Cholangiocarcinoma"[MeSH] OR "Bile Duct Neoplasms"[Mesh] OR "Klatskin's Tumor"[Mesh] OR cholangiocarcinoma*[tiab] OR klatskin tumor*[tiab] OR klatskin tumour*[tiab] OR HCCA[tiab] OR proximal bile duct tumor* OR extrahepatic bile duct tumor* OR proximal bile duct cancer* OR extrahepatic bile duct cancer* OR proximal bile duct tumour* OR extrahepatic bile duct tumour* OR proximal biliary cancer*) AND ("Laparoscopy"[MeSH Terms] OR laparoscop*[tiab]) AND ("Neoplasm Staging"[Mesh] OR staging[tiab]).

EMBASE: (exp bile duct carcinoma/ or exp bile duct tumor/ or Klatskin tumor/ or (cholangiocarcinoma* or klatskin tumor* or klatskin tumour* or HCCA or proximal bile duct tumor* or extrahepatic bile duct tumor* or proximal bile duct cancer* or extrahepatic bile duct cancer* or proximal bile duct tumour* or extrahepatic bile duct tumour* or proximal biliary cancer* OR bile tract carcinoma* OR biliary carcinoma* OR biliary duct carcinoma* OR biliary tract carcinoma*).ti,ab,kw.) AND (exp laparoscopy/ or laparoscop*.ti,ab,kw.) AND (cancer staging/ or staging/ or staging.ti,ab,kw.).

*Data collection and assessment of data quality*

Data will be extracted by two authors (R.J.S.C. and A.T.R.) using a spreadsheet. The following data will be collected: author and institution, publication date, study period, study design, number of patients undergoing laparoscopy, number of completed procedures, number of patients undergoing laparoscopic IOUS, number of avoided laparotomies, total number of patients with unresectable disease, number of liver, peritoneal and lymph node metastases, locally advanced disease, other reasons for unresectability, true positives (total number of unresectable cases), true negatives (all patients that undergo resection), false positives (always zero), false negatives (all patients that are unresectable at laparotomy), complications following SL and time interval between SL and laparotomy. Yield is defined as the total number of avoided laparotomies by the total number of laparoscopies. Accuracy (sensitivity) is calculated by dividing total number of avoided laparotomies by all patients with unresectable disease.

Previous reported series by the same institution in the same study period will be considered as duplicates and excluded. No attempt will be done to collect missing data.

The methodological quality will be independently assessed by both reviewers with the use of the Quality Assessment of Diagnostic Accuracy Studies (QUADAS-2) tool^[5]^. This tool is integrated in the RevMan software (Review Manager Version 5.3. Copenhagen: The Nordic Cochrane Centre, The Cochrane Collaboration, 2014).

Statistical analysis will be performed using StatsDirect Version 2.8.0 (StatsDirect statistical software. http://www.statsdirect.com. England: StatsDirect Ltd. 2013) for yield calculations and Meta-DiSc Version 1.4 (XI Cochrane Colloquium, Barcelona, 2006) for sensitivity calculations. Heterogeneity among the studies will be tested using Cochran’s Q-test and the amount of variation by heterogeneity will be reflected by the inconsistency index value (*I*^2^). Results of individual studies, when available, will be pooled and summary estimates are given using a random-effect model (DerSimonian-Laird). No funnel plots will be created in case of inclusion of a low number of studies.

**References**

1. Ito F, Cho CS, Rikkers LF, Weber SM. Hilar cholangiocarcinoma: current management. *Ann Surg* 2009;**250**(2): 210-218.

2. Matsuo K, Rocha FG, Ito K, D'Angelica MI, Allen PJ, Fong Y, Dematteo RP, Gonen M, Endo I, Jarnagin WR. The Blumgart preoperative staging system for hilar cholangiocarcinoma: analysis of resectability and outcomes in 380 patients. *J Am Coll Surg* 2012;**215**(3): 343-355.

3. Ruys AT, van Haelst S, Busch OR, Rauws EA, Gouma DJ, van Gulik TM. Long-term survival in hilar cholangiocarcinoma also possible in unresectable patients. *World J Surg* 2012;**36**(9): 2179-2186.

4. Moher D, Liberati A, Tetzlaff J, Altman DG. Preferred reporting items for systematic reviews and meta-analyses: the PRISMA statement. *BMJ* 2009;**339**: b2535.

5. Whiting PF, Rutjes AW, Westwood ME, Mallett S, Deeks JJ, Reitsma JB, Leeflang MM, Sterne JA, Bossuyt PM. QUADAS-2: a revised tool for the quality assessment of diagnostic accuracy studies. *Ann Intern Med* 2011;**155**(8): 529-536.
